# A weighted distance-based approach for deriving consensus tumor evolutionary trees

**DOI:** 10.1093/bioinformatics/btad230

**Published:** 2023-06-30

**Authors:** Ziyun Guang, Matthew Smith-Erb, Layla Oesper

**Affiliations:** Department of Computer Science, Carleton College, Northfield, MN 55057, USA; Department of Computer Science, Carleton College, Northfield, MN 55057, USA; Department of Computer Science, Carleton College, Northfield, MN 55057, USA

## Abstract

**Motivation:**

The acquisition of somatic mutations by a tumor can be modeled by a type of evolutionary tree. However, it is impossible to observe this tree directly. Instead, numerous algorithms have been developed to infer such a tree from different types of sequencing data. But such methods can produce conflicting trees for the same patient, making it desirable to have approaches that can combine several such tumor trees into a consensus or summary tree. We introduce The Weighted m-Tumor Tree Consensus Problem (W-*m*-TTCP) to find a consensus tree among multiple plausible tumor evolutionary histories, each assigned a confidence weight, given a specific distance measure between tumor trees. We present an algorithm called TuELiP that is based on integer linear programming which solves the W-*m*-TTCP, and unlike other existing consensus methods, allows the input trees to be weighted differently.

**Results:**

On simulated data we show that TuELiP outperforms two existing methods at correctly identifying the true underlying tree used to create the simulations. We also show that the incorporation of weights can lead to more accurate tree inference. On a Triple-Negative Breast Cancer dataset, we show that including confidence weights can have important impacts on the consensus tree identified.

**Availability:**

An implementation of TuELiP and simulated datasets are available at https://bitbucket.org/oesperlab/consensus-ilp/src/main/.

## 1 Introduction

Tumor progression has been recognized as an evolutionary process where somatic mutations accumulate (Nowell 1976), leading to the growth of a tumor. A tumor’s evolutionary history is the order and configuration in which these mutations arose. A better understanding of this history provides important insights into tumors’ development processes, such as the selection for variants that lead to tumor growth and tumor migration, which helps scientists develop more effective treatment plan for patients (Fittall and Van Loo 2019).

Many computational methods have been developed to derive the evolutionary histories of tumors, typically depicting them as rooted trees where the nodes represent tumor cell populations, and the edges indicate ancestral relationships (Schwartz and Schäffer 2017). There has been explosive growth in the methods that infer such a tumor tree from DNA sequencing data. For example, Jahn et al. (2016) and Malikic et al. (2019b) use single-cell data, Malikic et al. (2019a) uses both single-cell and bulk sequencing data, and Myers et al. (2019) uses longitudinal data. Different methods also consider different types of mutations (e.g. SNVs, CNAs, etc.) as input. Although advancements in different algorithms and methods can produce improved inference of tumor evolutionary histories, interpretation can be challenging due to the multiple possible trees returned from these methods, even when run on data from the same patient. It would be useful to combine these sets of output trees to better infer a single evolutionary history that best represents the tumor’s evolutionary process.

In recent years, several approaches have been introduced to identify a consensus tumor tree from a set of disparate input trees for a single patient. GraPhyC, first introduced in Govek et al. (2018) and then extended in Govek et al. (2020), is a graph-theoretic approach that aims to find a consensus tree with minimal total distance from all input trees for a specified tree distance function. However, there are several limitations to this method. First, it requires a pre-clustering step to identify a set of mutation clusters before identifying a consensus tree. This means that the approach is only able to consider a single mutation clustering. Second, this method is optimized for a specific distance measure called Parent–Child (PC) distance. While this distance measure is easy to compute, it has been suggested (DiNardo et al. 2019) that other distance measures may be more appropriate for comparing tumor evolutionary trees. Aguse et al. (2019) instead find multiple consensus trees, rather than a single tree. However, this approach still relies upon optimization for the PC distance as well. More recently, Fu and Schwartz (2021) used a different approach to the consensus tree problem which instead aims to find a tree that maximizes directed partition support from the input trees. This approach does consider different mutation clusterings, unlike GraPhyC and the method from Aguse et al. (2019), but its implementation only allows it to operate on trees which have nodes containing four or fewer children. Consensus approaches have also recently be used to detect evolutionary patterns across patients (Christensen et al. 2020).

One major downside to all existing tumor tree consensus methods is that they consider all input trees as equally informative. However, tumor evolution inference methods have been evolving quickly and make different assumptions or use varying techniques (Schwartz and Schäffer, 2017). Furthermore, there have been more studies where multiple types of sequencing data are available for a single patient (e.g. Gawad et al. 2014; Leung et al. 2017; and others). These studies can contain bulk, single-cell, and even longitudinal sequencing data, making features like the depth and quality of these different datasets even more important to consider when constructing consensus trees from them. For example, more weight could be given to trees constructed using the higher coverage data.

Here, we introduce an integer linear programming (ILP) based method to solve the consensus tree problem, but that allows for the input trees to have confidence weights. We name our method TuELiP (**Tu**mor **E**volution integer **Li**near **P**rogram). Specifically, we pose the Weighted *m*-Tumor Tree Consensus Problem (W-*m*-TTCP), which given (i) a set of input tumor trees, (ii) weights for each tree; and (iii) a tumor tree distance measure, finds a consensus tree that minimizes the total weighted distance from it to all input trees. In contrast to other existing distance-based consensus methods, our approach allows all clusterings of mutations to be considered when identifying a consensus tree, and optimizes for a more complex distance measure [Ancestor–Descendant (AD) distance]. To our knowledge, TuELiP is the first tumor consensus tree approach to allow for the input trees to be weighted differently. We validate our approach on both simulated and a Triple-Negative Breast Cancer (TNBC) dataset and show that our method outperforms two competing methods. On the real data, we show that including confidence weights can have important impacts on the consensus tree identified.

## 2 Materials and methods

### 2.1 Problem formalization

Let [m]={1,…,m} be a set of *m* mutations. A tumor that has acquired *m* mutations during its evolution, can be described using the following definition. An *m-tumor tree* is a directed rooted tree *T* where: (i) each node in the tree is labeled by one or more mutations from the mutation set [m]; and (ii) the collection of mutation labels over all nodes form a partition of the mutation set [m], which we call a “mutation clustering.” The nodes in *m*-tumor trees correspond to clones in a heterogeneous tumor where its cell population contains a unique set of somatic mutations. The direction of the edges indicate that a child clone originated from the cells of a parent clone. Thus, node labels represent the new mutations acquired by the clone which distinguish it from its parent, and are also the mutations that will be inherited by its descendants. It will also be useful to refer to the space of all *m*-tumor trees. Therefore, we define $T_{m}$ to be the set of all *m*-tumor trees. Finally, we also define l(v)⊆[m] to be the cluster of mutations labeling node *v*.

A recent problem of interest in the computational cancer field has been how to identify a consensus, or summary tree, from a set of conflicting, but similar tumor trees. Two general approaches to this problem have been proposed: (i) distance based methods (Govek et al. 2020, 2018); and (ii) partition based methods (Fu and Schwartz 2021). However, both types of approaches treat all input trees equally, and often restrict the trees considered to a subset of all possible *m* tumor trees. Our approach builds upon the distance-based methods, but allows for the input trees to be weighted rather than treated equally, and considers the entire space of *m* tumor trees $T_{m}$. We introduce the following problem.

**The Weighted *m*-Tumor Tree Consensus Problem (W-*m*-TTCP)**: Given a set $S=\{T_{1},T_{2},\ldots ,T_{n}\}\subseteq T_{m}$ of *m*-tumor trees, an *m*-tumor tree distance measure *dist*(.,.), and a tree weighting function w:S→[0,1] such that $\sum_{i=1}^{n}w(T_{i})=1$, find a consensus tree $T^{*}$ such that


(1) $$T^{*}=\underset{T\in T_{m}}{\text{arg}\;\text{min}}\sum_{i=1}^{n}w(T_{i})\cdot \mathrm{dist}(T,T_{i}).$$

We note that this formalization can easily be extended to find *k* consensus trees, as is done by Aguse et al. (2019). Our implementation of TuELiP also allows for the finding of all consensus trees that minimize the objective function.

### 2.2 Distance measures

Distance-based consensus methods require the use of a distance measure that considers how similar two input trees are, with lower values for more similar trees and higher values for more dissimilar trees. Previous distance-based consensus methods focused on one distance measure called PC distance (Govek et al. 2020). This distance measure counts the number of unique PC mutation relationships that appear in one tree but not the other. This distance measure has been used in previous consensus approaches mainly due to how easy it is to compute. However, it has been shown that other distance measures, in particular, those that consider not just parental but also longer range ancestral relationships may be more accurate (DiNardo et al. 2019). One reason for this is that ancestral mutations are inherited by all of their descendants, not just their children. Therefore, we will focus on using a distance measure called AD distance originally proposed by Govek et al. (2020) and described below.

For an *m*-tumor tree *T*, and distinct mutations *i* and *j*, we say *i* is ancestral to *j* if *i* labels a node which is on the path from the root to the node labeled by *j*. When *i* and *j* label the same node, they are considered ancestral to each other since their exact ordering is unknown. Additionally, if *i* is ancestral to *j*, we say that (*i*, *j*) is an “Ancestor-Descendant pair.” Given the *m*-tumor tree *T*, we define $\phi_{AD}(T)=\{(i,j)|$*i* is ancestral to *j* in *T*} to be the set of all AD pairs in *T*. Given two *m*-tumor trees *T*_1_ and *T*_2_, the AD distance between them is the number of AD pairs in one tree but not the other. Formally, the “Ancestor-Descendant (AD) distance measure” is defined as:


(2) $$AD(T_{1},T_{2})=|\phi_{AD}(T_{1})\setminus \phi_{AD}(T_{2})|+|\phi_{AD}(T_{2})\setminus \phi_{AD}(T_{1})|.$$

### 2.3 Our method

**Figure 1.**
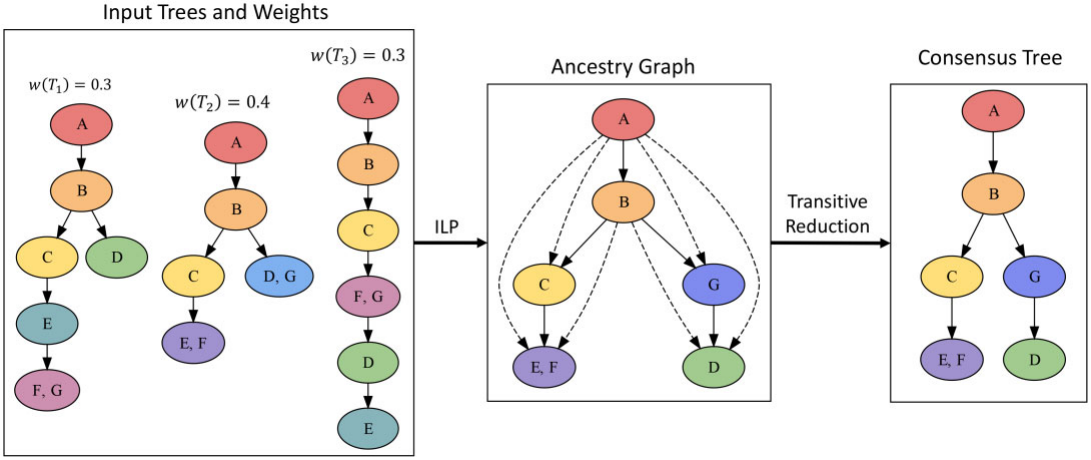
Overview of the TuELiP method. Consensus tree is found as a transitive reduction on ILP output graph, which has edges between nodes that are ancestral to each other in the final tumor tree.

We take a two-step approach to solving the W-*m*-TTCP when the distance measure is AD distance. (i) We first use an ILP to find a directed acyclic graph (DAG) whose nodes are labeled with mutations and whose edges represent all ancestral relationships between those mutations. We will show that this graph represents the transitive closure of the desired consensus tree. (ii) We then turn the resulting DAG into a directed tree through a transitive reduction. This is our consensus tree. Figure 1 shows an overview of our approach. In contrast to the existing methods (Govek et al., 2020; Fu and Schwartz, 2021), our approach considers all possible mutation clusterings (i.e. assignment of mutation labels to nodes), rather than restricting to a single mutation clustering, and utilizes a confidence weight assigned to each input tree when constructing the consensus tree.

#### 2.3.1 ILP variables and constraints

**Figure 2.**
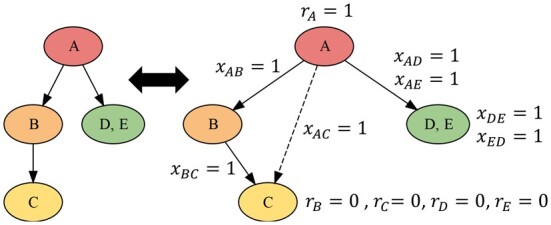
A small example on how the variables used in our ILP formulation might be set for a particular tree (*x_ij_* denoting ancestral relationship between mutations *i* and *j* and *r_i_* denoting whether mutation *i* labels the root). For example, since mutation B is ancestral to mutation C in the tree on the left, the variable *x_BC_* is set to 1.

We start by introducing two sets of variables that model the ancestral relationships in our solution. For every pair of unique mutations a,b∈[m] such that a≠b, we introduce a variable *x_ab_* where *x_ab_* = 1 if mutation *a* is ancestral to mutation *b*, and *x_ab_* = 0 if *a* is not ancestral to *b*. In the case if *a* and *b* label the same node, both *x_ab_* = 1 and *x_ba_* = 1. For each mutation a∈[m] we also introduce a variable *r_a_* where *r_a_* = 1 if mutation *a* labels the root of our consensus tree and *r_a_* = 0 otherwise. For a root with multiple labels, the corresponding set of variables are all set to 1. Figure 2 shows a small example of how these variables would be set for a specific *m*-tumor tree.

To ensure that these variables are set in a way that yields a configuration consistent with the transitive closure of a valid *m*-tumor tree, we introduce the following linear constraints.


(3) $$x_{ac}\geq x_{ab}+x_{bc}-1\;\;\forall a,b,c\in [m],a\neq b\neq c\neq a.$$

(4) $$x_{ab}+x_{ba}\geq x_{ac}+x_{bc}-1\;\forall a,b,c\in [m],a\neq b\neq c\neq a.$$

(5) $$\sum_{a\in [m]}r_{a}\geq 1.$$

(6) $$\sum_{b\in [m],b\neq a}x_{ab}\cdot \frac{1}{m-1}\geq r_{a}\;\;\forall a\in [m].$$

(7) $$\sum_{b\in [m],b\neq a}x_{ab}-(m-2)\leq r_{a}\;\;\forall a\in [m].$$

(8) $$x_{ab}\in \{0,1\}\;\;\forall a,b\in [m],a\neq b.$$

(9) $$r_{a}\in \{0,1\}\;\;\forall a\in [m].$$

We need to ensure that the ancestral relations output by TuELiP are transitive. That is, if mutation *a* is ancestral to mutation *b* and mutation *b* is ancestral to mutation *c* then we need to enforce that mutation *a* is ancestral to mutation *c*. This is accomplished with constraint (3). Furthermore, we need to ensure that if mutations *a* and *b* are both ancestral to *c*, then either *a* is ancestral to *b* or *b* is ancestral to *a*, or *a* and *b* are in the same node. This keeps all ancestral mutations on a single lineage path. Accordingly, constraint (4) ensures that if both *x_ac_* and *x_bc_* are 1, then at least one of *x_ab_* and *x_ba_* is also set to 1.

We also need constraints that ensure that the output from TuELiP is a connected DAG with a single root node. Constraint (5) ensures that there is at least one mutation in the root. To ensure the resulting graph is connected, constraint (6) requires that all mutations labeling the root are ancestral to all other *m −* 1 mutations (this includes any other mutations that also label the root). Similarly, constraint (7) ensures that any mutation that does not label the root must be ancestral to fewer than *m −* 1 mutations. Additional explanation of these constraints is provided in the Supplementary Appendix.

#### 2.3.2 ILP objective function

Recall we want to find the setting of TuELiP variables subject to our constraints that minimizes the total weighted AD distance from the set of input trees $\left\{T_{1},\ldots ,T_{n}\right\}$ to the resulting tree $T^{*}$ (whose transitive closure the ILP returns). To formulate this objective function using our variables, we introduce the function δ(T,a,b), which is 1 if mutation *a* is ancestral to mutation *b* in tree *T*, and 0 if *a* is not ancestral to mutation *b* in tree *T*. We show below how to formulate our desired objective function we wish to minimize.


$$\begin{array}{l} \sum_{i=1}^{n}\;w(T_{i})\cdot AD(T^{*},T_{i})\\ =\sum_{i=1}^{n}\;w(T_{i})\cdot \left[\left|\phi_{AD}(T^{*})\setminus \phi_{AD}(T_{i})|+|\phi_{AD}(T_{i})\setminus \phi_{AD}(T^{*})\right|\right]\\ =\sum_{i=1}^{n}\;w(T_{i})[\sum_{a,b\in [m]}x_{ab}(1-\delta (T_{i},a,b))+\sum_{a,b\in [m]}\left(1-x_{ab}\right)\delta (T_{i},a,b)]\\ =\sum_{i=1}^{n}\;w(T_{i})\sum_{a,b\in [m]}\left[x_{ab}(1-\delta (T_{i},a,b))+(1-x_{ab})\delta (T_{i},a,b)\right]\\ =\sum_{i=1}^{n}\;w(T_{i})\sum_{a,b\in [m]}\left[\delta (T_{i},a,b)+x_{ab}(1-2\delta (T_{i},a,b))\right] \end{array}$$

We note this objective function has a very natural interpretation. If the output tree does not have *a* ancestral to *b* given by the assignment of *x_ab_* = 0, but in a input tree *T_k_*, *a* is ancestral to *b*, then the objective function helps to add a penalization of $w(T_{k})$ to the objective. Conversely, if the output tree has *a* being ancestral to *b* given by *x_ab_* = 1, but in the input tree *T_j_ a* is not ancestral to *b*, then $\delta (T_{j},a,b)=0$ and a penalization of $w(T_{j})$ will be added to the objective.

#### 2.3.3 Transitive reduction from ILP solution

The final step for our approach is to convert the output from the ILP into an *m*-tumor tree. Specifically, we construct a DAG $G^{*}=(V^{*},E^{*})$ from the ancestral relationships indicated by the *x_ab_* variables and then create the *m*-tumor tree $T^{*}$ from $G^{*}$ by finding its transitive reduction. Creation of $G^{*}$ is straightforward. If $x_{ab}=x_{ba}=1$, then *a* and *b* label the same node in $V^{*}$. There is an edge from node *v* to node *w* if any mutation in *l*(*v*) is ancestral to any mutation in *l*(*w*). Because of the enforced transitive property of ancestral relationships [constraints (3) and (4)], it is sufficient to only consider a single mutation in a node when constructing edges. Formally, $E^{*}=\{(v,w)|x_{ab}=1,a\in l(v),b\in l(w)\;\text{where}\;v\neq w\}$. Finally, we perform a transitive reduction of $G^{*}$ (Aho et al. 1972). We then show that the output of the transitive reduction is guaranteed to be an arborscence and is the returned consensus tree.

### 2.4 Solving the *W*-*m*-TTCP

In order to show the efficacy of our approach to solving the *W*-*m*-TTCP when the distance measure is AD distance, we need to show that the output of our ILP, after a transitive reduction, is guaranteed to be an arborescence. An “arborescence” is a directed graph that contains a node called the root and that has a directed path from the root to all other nodes. First we make two observations (full proofs are provided in the Supplementary Appendix) that will be useful in showing that the output of our method fits this definition.

Observation 1. *The graph* $G^{*}=(V^{*},E^{*})$*produced by our ILP is acyclic.*

For a directed acyclic graph *G* with *n* nodes, there is a construction that finds a transitive reduction of *G* in $O(n^{2})$, denoted *G^t^*, which is unique and defined as having the following two conditions (Aho et al. 1972).

- There is a directed path from node *u* to node *v* in *G^t^* if and only if there is a directed path from *u* to *v* in *G*.
- There is no graph with fewer edges than *G^t^* satisfying condition (i).

Observation 2. *The transitive reduction of* $G^{*}$, denoted as ${\left(G^{*}\right)}^{t}$*, is acyclic.*

We can now prove the following theorem about the output of our method.

Theorem 1. ${\left(G^{*}\right)}^{t}$*is an arborescence.*

*Proof.* To prove ${\left(G^{*}\right)}^{t}$ is an arborescence, we must show that for a node *u*, called the root, there exists one path from *u* to any other node *v* (Gordon and McMahon 1989). We let *u* be the node that contains mutations that are ancestral to all other mutations. The existence of such a node is guaranteed by our constraints and can be identified using the *r_a_* variables from the ILP. Thus, there are paths from *u* to any other node *v* in $G^{*}$, which implies there exists a path from *u* to any other node *v* in ${\left(G^{*}\right)}^{t}$ per condition (i) above. Now, we need that there cannot exist more than one path from *u* to any node *v*.

Assume that there do exist multiple paths from *u* to *v*. Also since ${\left(G^{*}\right)}^{t}$ is acyclic (by Observation 2), there must exist a node on a path from *u* to *v*, say *w* (which may be *v*) that has more than one parent. Let *y* and *z* be two arbitrary and distinct parents of *w*. Then, let a∈l(y) and b∈l(z). By constraint (4), *a* is ancestral to *b* or *b* is ancestral to *a*, which implies that in $G^{*},\;(y,z)\in E^{*}$ or $(z,y)\in E^{*}$. Without loss of generality, say that $(y,z)\in E^{*}$, and so there must exist a path from *y* to *z* in ${\left(G^{*}\right)}^{t}$. So, there exist distinct paths from *u* to *v*, one that contains the edge (*y*, *w*), and one that contains the edge (*z*, *w*). By removing the edge (*y*, *w*) from *G^t^*, there still exists a path from *u* to *y*, *z*, *w*, and *v*, namely the path that went from *u* to *y*, to the edge (*z*, *w*), to *x*. Therefore, we have constructed a graph with fewer edges than ${\left(G^{*}\right)}^{t}$ that still follows property (i) of being a transitive reduction. This contradicts property (ii) that ${\left(G^{*}\right)}^{t}$ is the transitive reduction of $G^{*}$.

Thus, for any node *v* in ${\left(G^{*}\right)}^{t}$, there is exactly one path from the root *u* to *v*. This implies that ${\left(G^{*}\right)}^{t}$ is an arborescence. □

We have shown that the output of our method is an arboresence, a type of directed tree, and is therefore a valid *m*-tumor tree. Furthermore, we have shown that this tree minimizes the total AD distance to the set of input trees.

### 2.5 Different mutation sets

We have also modified our approach to work with input trees that contain different sets of mutations. In short, mutations are only present in the consensus tree if they occur in more than half of all the input trees. For more details, see the Supplementary Appendix.

## 3 Results

We implemented TuELiP in Python 3.7.1 and use the MIP library v1.13.0 (Santos and Toffolo 2020). We test the application of our method on both simulated and real data.

### 3.1 Simulated data

First, we describe our approach for simulated data creation. Second, we assess TuELiP’s ability to solve the W-*m*-TTCP problem. Third, we compare how well TuELiP does against other consensus models at uncovering the true simulated tree of a patient. Finally, we analyze the effects of using different weighting schemes on the input trees.

#### 3.1.1 Dataset creation

We simulate sets of input trees with a method similar to (Fu and Schwartz 2021). We create 6 datasets, each composed of 100 trials over all combinations of 5 or 10 input trees and 10, 20, or 30 mutations per tree. For each trial, we generate a ground truth tree with three steps: (i) randomly cluster mutations such that the expected number of clusters/nodes is $1+\frac{3}{4}(m-1)$, (ii) randomly assign parental relationships to each cluster such that all clusters have three or fewer children, (iii) assign mutation frequencies to each cluster while adhering to the sum rule (a cluster’s frequency must be greater than or equal to the sum of its children’s frequencies) (Jiao et al. 2014). We create each input tree for the given trial with the following modifications to the ground truth tree. Each modification is applied with the given probability during a traversal of all nodes in the tree, as long as the sum rule is still followed: (i) move the subtree rooted at each node with a probability of 1/3 to a grandparent or sibling node (ii), collapse edges between nodes with a probability inversely related to the closeness in mutation frequencies between the two nodes, (iii) randomly expand multi-labeled nodes with a constant probability of 1/3 into a new parental node and a child node. During the input tree generation process, we also ensure that no tree has a branching factor exceeding four, which allows us to run the consensus method ConTreeDP (Fu and Schwartz 2021) on the simulated trees. For full details, see the Supplementary Appendix.

#### 3.1.2 Ability to solve W-m-TTCP

We evaluated the efficacy of TuELiP at solving the W-*m*-TTCP compared to GraPhyC’s approach (Govek et al. 2020). We do not evaluate ConTreeDP in this scenario because its output is not a median tree, that is, it is not trying to minimize the total distance to the input trees. Instead, ConTreeDP creates an output tree that maximizes directed partition support from the input trees (Fu and Schwartz 2021), and this formulation does not yet have a corresponding tumor tree distance. For this experiment, we run both our method and GraPhyC on all six simulated datasets and calculate the total distance from each method’s consensus trees to all input trees for each trial using PC distance (which GraPhyC optimizes for), AD distance (which TuELiP optimizes for), as well as two other tumor evolution tree distances: CASet and DISC (DiNardo et al. 2019).

**Figure 3.**
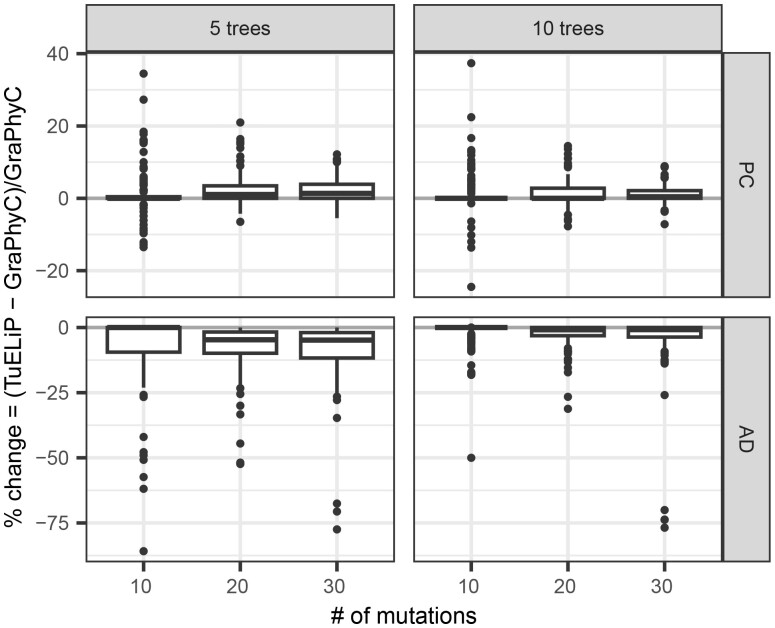
A boxplot showing how well our approach solves the W-*m*-TTCP problem for PC distance, AD distance in terms of percent change from the output found by GraPhyC. A negative value indicates that our consensus tree is closer to the input trees than GraPhyC’s consensus tree.

Since GraPhyC does not consider weights for input trees, we weigh all trees equally in this experiment so we can make fair comparisons. For each trial and distance measure, we calculate the percent change of the total distance between GraPhyC and TuELiP, in order to show the differences in outputs of these two methods directly. We define the percent change from GraPhyC as the difference of TuELiP’s total distance to the input trees and GraPhyC’s total distance, all divided by GraPhyC’s total distance to the input trees. Thus, 0% indicates their consensus trees were equally close to the input trees, a −50% change would indicate TuELiP’s consensus tree had a total distance to the input trees that is 50% less than the distance from the output of GraPhyC to the input trees, and a 50% change indicates that TuELiP’s tree had a total distance to the input trees that was 50% greater than the output of GraPhyC. Figure 3 shows our results for PC and AD distances. The results for CASet and DISC are similar to AD and are included in the Supplementary Appendix.

We see that our approach always returns a solution closer to, or equal to, all input trees in terms of AD distance (negative percent changes), while GraphyC usually (85% of trials) outperforms or ties with regards to PC distance. It is not surprising that TuELiP outperforms GraPhyC when considering AD distance, as our method is designed to optimize for that distance measure. However, it is notable that we still perform better than GraPhyC in some situations when considering PC distance, which GraPhyC is optimized for. Our method also has a smaller AD percent change when there are 10 input trees versus 5 input trees. We see similar trends to AD when using CASet and DISC to measure percent change. Specifically, TuELiP outperforms or ties GraPhyC in 94% trials when using CASet and in 91% trials when using DISC. Thus, TuELiP performs well under a variety of distance measures.

#### 3.1.3 Ability to uncover the true tree

To demonstrate TuELiP’s ability to uncover the true tree used to create each simulation, we compare its performance to two other consensus methods: GraPhyC (Govek et al. 2020) and ConTreeDP (Fu and Schwartz 2021). Since neither of these methods have the ability to consider weights for the input trees, we set the weights for our method to be equal for all trees.

**Figure 4.**
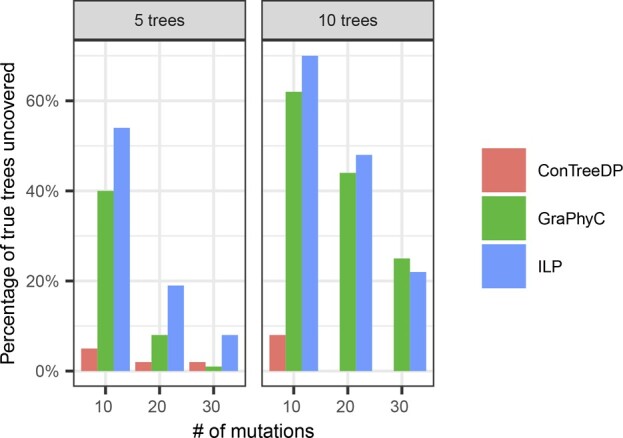
The results of counting the proportion of trials in which each method returned the exact true tree.

We ran all three models on the 600 simulated trials and counted the number of trials in which they returned the exact ground truth tree. Figure 4 shows that TuELiP uncovers more trees than the other methods for 5 input trees. However, for 10 input trees, TuELiP only uncovers more trees than GraPhyC for 10 and 20 mutations, and uncovers slightly fewer trees for 30 mutations (25% of true trees uncovered for GraPhyC and 22% for TuELiP).

On all simulations for all models we also calculate the distance from their found consensus tree to the ground truth tree. This allows for a more nuanced analysis of how well the methods perform. We use three different distance measures in this evaluation: (i) AD distance; (ii) CASet distance (DiNardo et al. 2019); and (iii) DISC distance (DiNardo et al. 2019). Unlike previous sections, we did not use PC distance here because the distance measure was only used to compare to GraPhyC and does not represent longer range ancestral relationships within tumor trees. DiNardo et al. (2019) also showed that PC distance has limited uses when comparing tumor trees.

For trials with 5 input trees, the median distance from our TuELiP’s consensus tree to the ground truth tree was lower than the median distances of both ConTreeDP and GraPhyC across all three distance metrics for 10, 20, and 30 mutations in each tree. Restricting to the 300 trials with 5 input trees, TuELiP found a tree that was closer to the ground truth tree than the tree found by ConTreeDP in around 220 trials using AD, CASet, and DISC. For the same 300 trials with 5 input trees, TuELiP found a tree closer to the true tree than the output of GraPhyC in around 160 trials with the three distance metrics. When there were 10 input trees, TuELiP consistently outperformed ConTreeDP by outputting trees closer to the true tree in at least 244 trials using the three distances, and TuELiP outperformed GraPhyC in at least 100 trials. In the trials with 10 input trees, there were many more ties between TuELiP and GraPhyC in comparison to the trials with 5 input trees. There were 48 more ties in the trials with 10 trees versus 5 trees using AD, 47 more ties using CASet, and 47 more ties using DISC.

**Figure 5.**
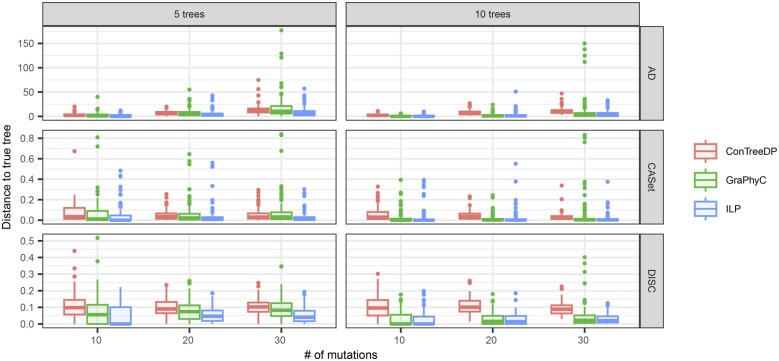
Results showing how well ConTreeDP, GraPhyC, and TuELiP uncover the true tumor trees for our simulated data. We measure the distances between the consensus trees and true trees using AD, CASet, and DISC.

We observe that while GraPhyC identifies more consensus trees correctly than TuELiP when there are 10 input trees, each with 30 mutations (Fig. 3), it has a higher mean distance to the true tree (11.09 using AD) than TuELiP (4.94 using AD) in these same trials (Fig. 5). This means that while GraPhyC is able to find the correct tree slightly more of the time for this particular dataset, when it gets it wrong, it is significantly more different from the correct tree than that inferred by TuELiP.

#### 3.1.4 Impact of input tree weighting

Our model allows a user to weight the input trees based on their level of confidence in each. Existing methods ConTreeDP or GraPhyC do not have this capability, so in our previous experiments we weighted all trees equally in order to be able to compare to these methods. In this section, we show how our method performs under different weighting schemes and show that this feature allows us to find improved consensus trees.

To test the effect of different weights on input trees, we try various weighting schemes and compare our distances to the true tree used to create the simulation. For each trial in our simulated data, we rank the input trees in ascending order of AD distance to the true tree of the trial. We use this to first create two baseline weighting schemes. The “Naive” weighting scheme assigns a weight of 1 to the tree closest to the input and a 0 to the other inputs, forcing the consensus tree to be the one closest tree. The “Constant” scheme refers to weighting all trees the same, as was done in the previous benchmarking. Finally, we model how a user might use weights by creating the “Linear” scheme where the closest tree was given the highest weight (0.3 for trials with 5 trees, 0.15 for 10 trees), and then the weight of each ranked tree decreased by a constant amount (0.05 for trials with 5 trees, 0.0111 for 10 trees). This weighting scheme is intended to mimic how a user may use outside information (e.g. sequencing coverage and type) to weight input trees. Table 1 shows the mean AD distance from the outputs of the three weighting schemes to the true tree.

**Table 1.** The mean AD distance from the output of TuELiP when using “Naïve,” “Constant,” and “Linear” weighting schemes on the input trees.^a^

No. of mutations	No. of trees	“Naive” weighting	“Constant” weighting	“Linear” weighting
10	5	7.45	1.92	**1.28**
10	10	6.05	0.94	**0.34**
20	5	18.88	5.43	**3.87**
20	10	21.28	3.07	**1.97**
30	5	39.24	**9.07**	9.26
30	10	39.94	4.94	**3.51**

a The values in bold indicate they are the lowest mean AD distance of the three weighting schemes. See the Supplementary Appendix for a boxplot of each trials’ distances.

The “Linear” scheme outperformed the “Naive” weighting scheme across trials with 5 and 10 input trees and 10, 20, and 30 mutations. “Linear” also outperformed “Constant” for all cases except 5 trees and 30 mutations, in which its mean distance was 2% higher than the mean distance of the “Constant” consensus trees. For the other cases, the “Linear” weighting scheme had a mean distance of at least 28% less than the mean distance of “Constant”. Additionally, out of all 600 trials, the “Linear” weighting scheme uncovered the true tree in 249 trials, compared to the “Constant” scheme uncovering the tree in 221 trials, and “Naive” uncovering the true tree in just 9 trials. This demonstrates the possible benefit of being able to use weights when identifying consensus trees. We also note that across all of the simulated data trials, the “Naive” weighting scheme significantly performed the worst. This further demonstrates the utility of finding the consensus of several possible trees instead of trying to select the single best input.

### 3.2 Real data

#### 3.2.1 TNBC dataset

We apply our method to a single-cell TNBC dataset from Wang et al. (2014). Karpov et al. (2019) applied three different single-cell sequencing tumor evolution inference methods, SCITE (Jahn et al. 2016), SiFit (Zafar et al. 2017), and PhISCS (Malikic et al. 2019b) to this dataset to recover three possible tumor evolution trees for the patient. Both GraPhyC (Govek et al. 2020) and ConTreeDP (Fu and Schwartz 2021) used this dataset in their original analysis after restricting the set of trees to the same set of 19 mutations that appear in all three trees. We use that same restricted dataset for our analysis here.

#### 3.2.2 Equal weights consensus tree

**Figure 6.**
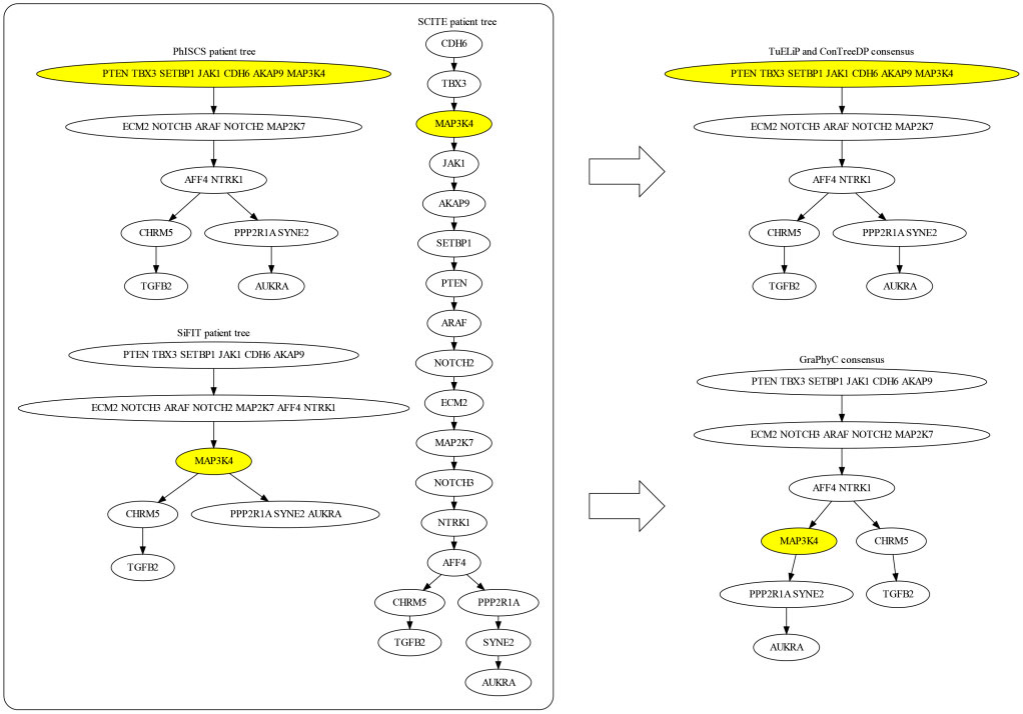
(Left) Each tumor tree was inferred by a separate method for a single TNBC patient. (Right) Two consensus tree were generated by TuELiP (and ConTreeDP) and GraPhyC. The nodes with yellow denote that they are labeled by mutation MAP3K4, the mutation whose placement differs between the two consensus trees.

We first apply TuELiP GraPhyC, and ConTreeDP to this TNBC dataset using constant weights for all three input trees. The input trees and inferred trees by all three methods are shown in Fig. 6. TuELiP and ConTreeDP generate identical consensus trees, which is the same as the tree inferred by PhISCS. The difference between this consensus tree and the one generated by GraPhyC is the placement of the mutation to the *MAP3K4* gene. Our method and ConTreeDP have it in a mutation cluster in the root with six other mutations, indicating it is a relatively early mutation in this history of this tumor, which is congruent with both the PhISCS and SCITE patient trees. GraPhyC pulled the mutation out from the root mutation cluster and made it a child of *AFF4* and *NTRK1*, indicating that it was a later mutation, which is only reflected in the SiFIT tree. Furthermore, the total distance from our (and ConTreeDP’s) consensus tree to the three input trees is lower than the distance from GraPhyC’s consensus tree to the input trees when using AD (24% lower), CASet (17% lower), and DISC (18% lower) distances. One reason that GraPhyC likely outputs this different tree is because it only considers a single mutation clustering, and is therefore forced to put *MAP3k4* in its own node. Both our method and ConTreeDP do not have this limitation and in fact are able to consider all possible mutation clusterings, leading to placing *MAP3k4* on a node with other mutations higher in the tree, which also is a better solution in terms of AD distance.

#### 3.2.3 Varied weights consensus trees

**Figure 7.**
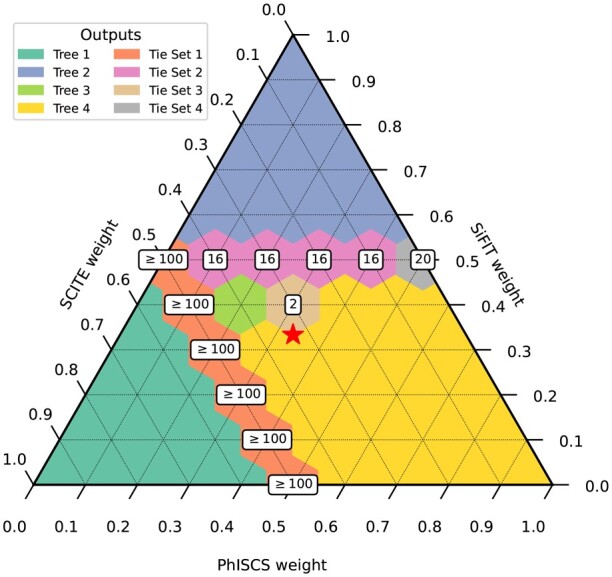
The weights of each input tree from (Karpov et al. 2019) were varied, while still adding up to 1, and used as input to TuELiP. Tree 1 (cyan) is the SCITE tree, Tree 2 (blue) is the SiFIT tree, and Tree 4 (yellow) is the PhISCS tree. The number of ties are present when there is more than one optimal tree for the given weight. All of the tree topologies can be found in the Supplementary Appendix. The red star denotes the center of the triangle in which all three trees were given a weight of $\frac{1}{3}$.

We also performed experiments on this TNBC dataset to show the impact of varying the confidence weights in the input trees when inferring a consensus tree. We varied the weights of each patient tree to cover all permutations of three numbers between 0 and 1 (with one decimal place) that sum to 1. The full resulting outputs can be seen in the ternary graph in Fig. 7 that indicates which tree, or trees, were output for each weighting configuration. As one might expect, the three corners of the plot, where one tree is much more heavily weighted than the other two, are dominated by the input trees found by SCITE, SiFIT, and PhISCS (labeled as Trees 1, 2 and 4, respectively, in the plot). Furthermore, the extent of these regions demonstrate that if the confidence weight for an individual tree exceeds 0.5 (meaning it exceeds the confidence of the other two trees combined), our method will output that tree. We also see two stripes across the plot where multiple solutions are found. The orange stripe (Tie Set 1) with greater than 100 solutions shows when we move away from the highly weighted SCITE tree (which puts many mutations in their own cluster) and start clustering the mutations, we obtain trees more similar to the other trees.

We also see that if we weight all trees equally (marked with a star in the plot), we return Tree 4 (the same as the input tree from PhISCS). The region associated with this tree is larger in the ternary plot than the regions associated with the other two input trees. However, the exact consensus tree(s) returned can change as these weights change. For example, if we use the weights w(PhISCS)=0.3, w(SiFIT)=0.4, and w(SCITE)=0.3, which more strongly support the SiFIT tree, we actually find that there are two optimal consensus trees, labeled as Tie Set 3 in the figure. This set includes the tree returned when using equal weights (Tree 4 in the plot) as well as an additional tree (Tree 3 in the plot) that is identical to this tree except that the mutation to *MAP3K4* labels its own node inserted in-between the nodes labeled by *PTEN, TBX3,…, AKAP9* and by *ECM2, NOTCH3,…, MAP2K7*. Both trees support this mutation as having occurred earlier than what was predicted by GraPhyC, but provides additional information on the uncertainty of when exactly this mutation occurred. This experiment highlights the important variations that may occur in inferred consensus trees when outside information, such as different types of sequencing data being used to infer different trees, allows for applying weights to these trees.

This plot also shows other interesting features of the space of consensus trees, which thus far have not been able to be captured by other consensus methods that do not consider weights for the input trees. For instance, we can see that a set of 16 equally scoring trees separate the regions between the PhISCS tree (Tree 4) and the SiFIT tree (Tree 2). A similar boundary region, but with even more trees exists between the regions dominated by the PhISCS tree (Tree 4) and the SCITE tree (Tree 1). However in this case, we only were able to find 100 trees in each set before timing out. Having the ability to explore the space of consensus trees, or even to be able to see how quickly or slowly the consensus tree changes when changing weights will provide additional information to support inferred consensus trees.

Two additional weighting experiments on trees inferred by Malikic et al. (2019a) are located in the Supplementary Appendix. These datasets include trees inferred from both bulk-sequencing and single-cell sequencing data.

## 4 Discussion

In this work we introduce the Weighted *m*-Tumor Tree Consensus Problem (W-*m*-TTCP), which given (i) a set of input tumor trees, (ii) weights for each tree, and (iii) a tumor tree distance measure, finds a consensus tree that minimizes the total weighted distance from it to all input trees. We then present an ILP method, TuELiP, that solves the W-*m*-TTCP when the distance measure is AD distance. In contrast to the existing distance-based consensus methods, TuELiP is able to consider all possible mutation clusterings when identifying a consensus tree and is optimized for a more appropriate distance measure (existing methods use the simpler PC distance). Furthermore, in contrast to all existing tumor tree consensus methods, TuELiP allows a user to weight different input trees differently based upon any outside knowledge they have about either the methods used to create those trees, or the data from which they were derived.

On simulated data we show that TuELiP is able to find better solutions to the W-*m*-TTCP when the distance measure is AD distance and all trees are weighted equally than the method GraPhyC. We also show that TuELiP is better at recovering the true underlying tree used to create the simulated data than both GraPhyC and ConTreeDP. On a real TNBC dataset, we found that TuELiP returned the same consensus tree as ConTreeDP which better represents input trees when equal weights were used. However, on this same dataset, we saw that variations in the confidence weights of the input trees could lead to alternative consensus trees being found—thus indicating the impact of incorporating weights into the consensus model.

There are a number of different methodological extensions we hope to make to this work. Our work here focuses on using the AD distance. While this is an improvement from previous work that used the simpler PC distance, there are other more specialized distance measures (e.g. DiNardo et al. 2019; Karpov et al. 2019; Ciccolella et al. 2021; Jahn et al. 2021) that might be even more appropriate to use. We would also like to explore the solution space of the W-*m*-TTCP using a generalization of the approach we took in Fig. 7. This would help to discover if concrete things can be said about when multiple optimal solutions exist for a given input, or if different patterns exist for how consensus trees change with weights.

Additionally, while our model presents a step forward as it allows all input trees to be weighted differently, the best way to choose such weights is not straightforward. We hope to do additional work to show how different features of the input data (e.g. inference algorithms used, sequencing type and coverage, etc.) can affect the inferred consensus tree and may be used when choosing weights.

## Supplementary Material

btad230_Supplementary_Data
