# Supplementary material for: A weighted distance-based approach for deriving consensus tumor evolutionary trees
Source: Bioinformatics. 2023 Jun 30;39(Suppl 1):i204–12. doi: 10.1093/bioinformatics/btad230 (PMC10311318; doi:10.1093/bioinformatics/btad230)
Supplement: btad230_Supplementary_Data [file btad230_supplementary_data.pdf]

Appendix: A Weighted Distance-Based Approach for Deriving Tumor Evolutionary Trees

Weighted m-Tumor Tree Consensus Problem

If there is a preexisting weighting scheme for the input trees, say $w'(.)$, in which the sum of the weights of the trees don't add to one, we can normalize them by defining $w(.)$ as follows.

$$w(T) = w'(T)/\sum_{i=1}^{n} w'(T_i), \forall T \in \mathcal{S} \quad (1)$$

Additional ILP Details

We include here additional examples explaining some of the ILP constraints. In particular we consider the following constraints from the main manuscript.

$$\sum_{b\in[m],b\neq a} x_{ab} \cdot \frac{1}{m-1} \geq r_a \qquad \forall a \in [m] \quad (6)$$

$$\sum_{b\in[m],b\neq a} x_{ab} - (m-2) \leq r_a \qquad \forall a \in [m] \quad (7)$$

We will now describe a situation where all constraints from the original ILP are satisfied except for constraint (6). Consider a small graph with 4 nodes ($m = 4$), labeled A,B,C,D. Only two edges exist in this graph, (A, B) and (C, D). Using this example we set values for the X and R variables. The only non-zero variables are as follows: $x_{AB} = 1$, $x_{CD} = 1$, $r_A = 1$, and $R_C = 1$. All other variables are set to 0. These settings satisfy all constraints except for constraint (6). Consider mutation A. If we sum all X variables out from A, there is only 1 that is set. So we have $\sum_{b\in[m]} x_{Ab} \cdot \frac{1}{m-1} = 1 \cdot \frac{1}{4-1} = \frac{1}{3} < 1 = r_A$. Hence, constraint (6) is not satisfied for mutation A.

We will now describe a second situation where all constraints from the original ILP are satisfied except for constraint (7). Consider a small graph with 3 nodes ($m = 3$), labeled A,B,C. Three edges exist in the graph, (A, B), (B, C), (C, A), making a cycle. Furthermore, node A is considered the root. The only variables set to 0 are as follows: $r_B = 0$ and $r_C = 0$. All other variables are set to 1 (including all x variables). These settings satisfy all constraints except for constraint (7). Consider mutation B. If we sum all x variables out from B, there are 2. So we have $\sum_{c\in[m]} x_{Bc} - (m-2) = 2 - (3-2) = 2 - 1 = 1 > 0 = r_B$. Hence, constraint (7) is not satisfied for mutation B.

Additional Proofs

Observation 1 The graph $G^* = (V^*, E^*)$ produced by TuELiP is acyclic.

Proof For a proof by contradiction, say there exists a cycle within G^* that contains distinct nodes v and w. Because E^* is transitive, there must exist edges (v, w) and (w, v). However, for mutations $a \in l(v)$ and $b \in l(w)$, edge (v, w) implies that $x_{ab} = 1$ and edge (w, v) implies that $x_{ba} = 1$. By the definition of V, a and b must label the same node, creating a contradiction. □ □

Observation 2 $(G^*)^t$ is acyclic.

Proof Since G^* is acyclic, there cannot exist any paths from a node v back to itself. This implies no such paths can exist in $(G^*)^t$. Thus $(G^*)^t$ cannot have any cycles. □ □

Further TuELiP Implementation Details

Different Mutation Sets

When there are different mutation sets across the input trees, we have our consensus tree only contain the mutations that are present in weighted majority of trees. As a result, we re-define $\delta(T, a, b)$ to be 1 if a is ancestral to b in tree T (or they're in the same node), and 0 if a is not ancestral to b or a or b are not mutations present in T.

Multiple Median Trees

There may exists multiple trees that minimize the total weighted AD distance to the input trees. Our implementation allows one to find and output more than one solution if there exist ties. This is done by first finding one solution and recording its optimal objective function value. We then add a constraint to the ILP such that at least one variable from the previous solution is set to a different value. Next, we solve the ILP again, and if the new objective function value is no worse than the previous one, we add it to the list of solutions. We repeat this process until the optimal solution has a higher distance to the true trees than the previous solutions, or we timeout at finding 100 solutions.

Simulated Dataset Creation

We simulate a true m-tumor tree with a max branching factor of three (no node can have more than three children) and assign mutations frequencies to its nodes adhering to the sum rule (Jiao *et al.*, 2014) with the following steps:

1. cluster the mutations 1,2,...,m:
   a. shuffle the mutations 1,2,...,m into a new list
   b. iterate through each mutation in the shuffled list, and with a fixed probability of 0.75:
      (i) place the mutation into a cluster by itself
      (ii) otherwise, with probability of 0.25, place the mutation into an existing cluster
2. assign random parents between the clusters:
   a. create a node from the first cluster in the list and set it to be the root
   b. iterate through all other clusters:
      (i) create a node from the given cluster
      (ii) set its parent to be an existing node in the tree, provided that the existing node has no more than two children
3. assign frequencies to each node representing how often all of the mutations in the clone appeared in the original sampling data:
   a. set the root node to have a frequency of 1
   b. iterate over all nodes in breadth-first order, for each node:
      (i) randomly sample positive numbers which sum to the node's frequency
      (ii) the size of the sample is the number of children plus 1
      (iii) assign all but one of the sampled frequencies to the children

Given a simulated true tree with assigned mutation frequencies adhering to the sum rule, we generate sample input trees from this true tree by making a copy of it, and then performing the following perturbations in order: move subtrees, collapse parent/child nodes, expand clusters. It should be noted that although the max branching factor of the true trees is three, we allow the max branching factor of the sample trees to be four to create more variation. We limit it to four to allow ConTreeDP (Fu and Schwartz, 2021) to be ran on these inputs for benchmarking of our own method.

We apply the first two perturbations to the ground truth tree by traversing it in reverse breadth-first and performing the edits to nodes with fixed probabilities. Due to the limitations from the sum-rule and max number of children, the perturbations which occur first will affect which later edits are possible. We found that applying the first two perturbations starting at the tree's leaves and then moving towards the root lead to greater variation at the bottom, and less variation near the top. Although these edits can change the ordering of nodes within the tree, we still visit each node in the traversal order based on the topology of the tree before the set of perturbations. For example, we visit each node in the reverse breadth-first order of the true tree during our perturbation which moves subtrees.

Randomly move subtrees

1. visit each node in the true tree with reverse breadth-first order, skipping those without a grandparent
2. with fixed probability 1/3, attempt to move the subtree rooted at the given node:

   a. move the subtree to be the child of a valid node which is defined to be either its grandparent, or any sibling such that the sum rule is still adhered to, and the new parent doesn't have more than 4 children (50/50 chance to attempt a move to its grandparent or any sibling)
   b. if an attempt is made but stopped because it violates the sum rule or branching factor, move on to moving the next node

Collapse parent and child nodes:

1. visit each node of the previously perturbed tree with reverse breadth-first ordering
2. for each child of the given node, provided that the number of children of the node plus the number of children of the child node does not exceed three, the probability of collapsing the child into the node is:

   a. (1 - ((node.frequency - child.frequency) / node.frequency) / node.frequency) * 0.5
   b. the closer the child's frequency is to its parent, the higher the probability of collapsing

Table 1. We compare and contrasted some aspects of current methods solving the tumor tree consensus problem.

Methods	TuELiP	GraPhyC	ConTreeDP	Aguse *et al.* (2019)
**Problem Solved**	W-m-TTCP	m-TTCP	†MDPSCT	‡MCT
**Distance Measure Optimized**	AD	PCD	Directed partitions	PCD
**Types of Algorithm**	Integer linear programming	Graph-based	Dynamic programming	Mixed integer linear programming
**Computation Time**	*3.8 seconds	*1 seconds	*1.2 seconds	**In seconds

†**MDPSCT** - Maximum Directed Partition Support Consensus Tree Problem;
‡**MCT** -Multiple Consensus Tree
***Computation time** of TuELiP and ConTreeDP (Fu and Schwartz, 2021) are the average time taken to run on 5 input trees each with 20 mutations, on Dell Poweredge 540 Server with 28 cores and 384 GB of RAM. Computational time of GraPhyC (Govek *et al.*, 2020) is the time taken to run on 4 input trees each with 16 mutations given from their example data, on the same server.
****Computation time** of the method from Aguse *et al.* (2019) to solve MCT is taken from their paper where it is reported as fractions of seconds.

Table 2. Comparison of advantages of methods solving the tumor tree consensus problem.

Methods	Advantages
TuELiP	- Consider all clusterings *<br>- Allows confidence weights on inputs<br>- Uses AD which emphasizes longer range evolutionary process<br>- Allows inputs trees to have different sets of mutations
GraPhyC	- Optimizes for PCD<br>- Outputs a single optimal solution
ConTreeDP	- Considers all clusterings *<br>- Outputs a single optimal solution
Aguse *et al.* (2019)	- Summarizes solution spaces with distinct topological features<br>- Heuristic approach recovers optimal solutions only at a fraction of time

*Consider all clusterings means consider complicated combinations of mutation types. Identify optimal trees by considering all ways of clustering of mutation labels.

3. to collapse, add the child's labels to the given node's labels, and all of the child's children to be children of the current node

Expand multi-labeled clusters:

1. visit each node of the previously perturbed tree in breadth first order
2. if the node has multiple mutations, and with fixed probability 1/3, expand the node:

   a. randomly select a subset of the mutations to be the top node, and the rest of the mutations are the bottom node
   b. set the children of the current node to be the children of the bottom node
   c. set the bottom to be the child of the top, and the top is the child of the parent of the original node
   d. reassign frequencies to the top and bottom nodes:

      (i) randomly sample two numbers between and upper limit and lower limit, such that they adhere to the sum rule
      (ii) the upper limit is calculated to be the original node's frequency minus the sum of the node's siblings' frequencies (or 1 if the original node is the root)
      (iii) the lower limit is calculated to be sum of the frequencies of the original node's children (0 if the original node has no children)
      (iv) assign the larger of the two random numbers to be the top node's frequency and the smaller number to be the bottom node's frequency

Additional Results

Solving the W-m-TTCP

We present in Fig. 1 the boxplots showing how TuELiP solves the W-m-TTCP for Parent Child (PC) distance, Ancestor Descendant (AD) distance, CASet and DISC (DiNardo *et al.*, 2019) in terms of percent change from the output found by GraPhyC.

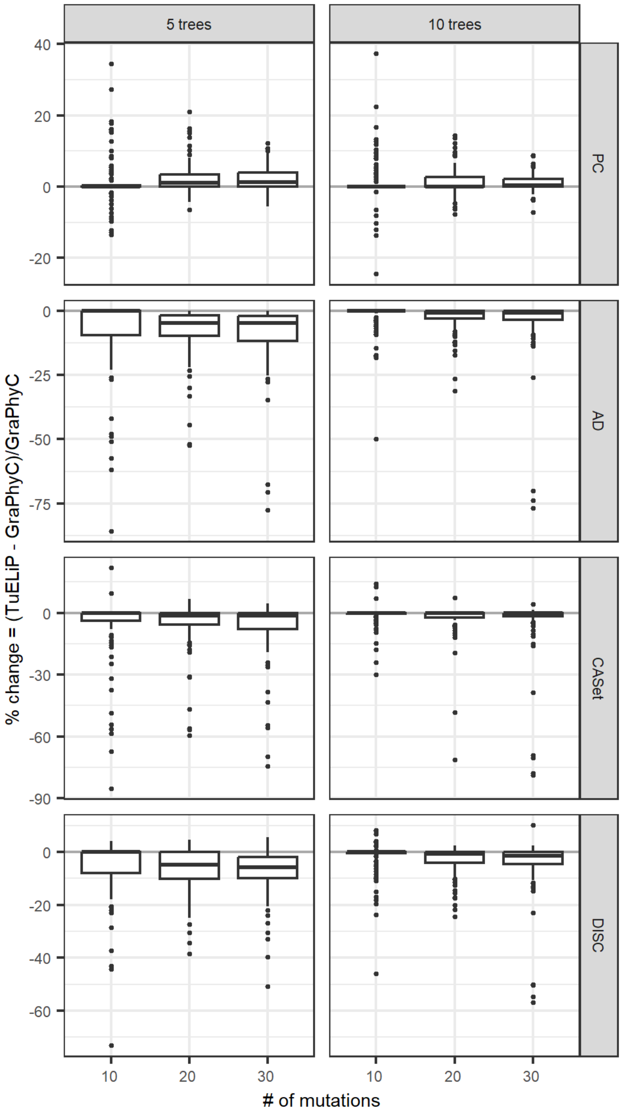

Fig. 1. A boxplot showing how well our approach solves the W-m-TTCP problem for Parent Child (PC) distance, Ancestor Descendant (AD) distance, CASet and DISC in terms of percent change from the output found by GraPhyC. A negative value indicates that our consensus tree is closer to the input trees than GraPhyC's consensus tree.

Weighting Schemes

We present the boxplots displaying the distances to the true tree given different weighting schemes on simulated data in appendix fig. 2.

Trees Inferred From TNBC Data

Figures 3-10 in this Appendix represent various trees and tree sets indicated in Figure 7 of the Main manuscript Some of the trees come from a larger set of trees which all had the optimal weighted distance to the inputs.

Additional Triangle Plots

We performed additional experiments to test how the weighting of patient-based tumor trees affected the output of TuELiP. The goal of these specific experiments was to try using input trees which were inferred from different types of sequencing data. We used the trees from two different studies, one on a patient with Acute lymphoblastic leukaemia (ALL) performed by Gawad *et al.* (2014) and another on a patient with Triple-negative breast cancer from a different study done by Wang *et al.* (2014). Malikic *et al.* (2019a) inferred three different trees for both sets of studies. They inferred a tree from the bulk sequencing data of each patient, another tree was inferred with SCITE (Malikic *et al.*, 2019b) using single-cell sequencing data, and the third tree was inferred using B-SCITE (the novel inference method proposed in their research) using both bulk and single-cell sequencing data. For each patient, we use their respective three inferred trees as inputs to TuELiP, and vary the weights of each input such that the total sums to 1.

As seen in Figure 11 (left), there is a single tree that is returned for when the weights of all three input trees are less than 0.5. The plot also has a similar appearance to our first TNBC ternary plot in the main paper, where there are many ties on the borders where a single tree has a weight of exactly 0.5, and the weights of the other two trees sum to 0.5. The primary difference is the center region is comprised of a tree that isn't present in the input trees. Additionally, this center region is even more homogeneous than the first TNBC ternary plot, where there are no ties in the middle where all trees are weighted less than 0.5. Despite all three trees having slightly

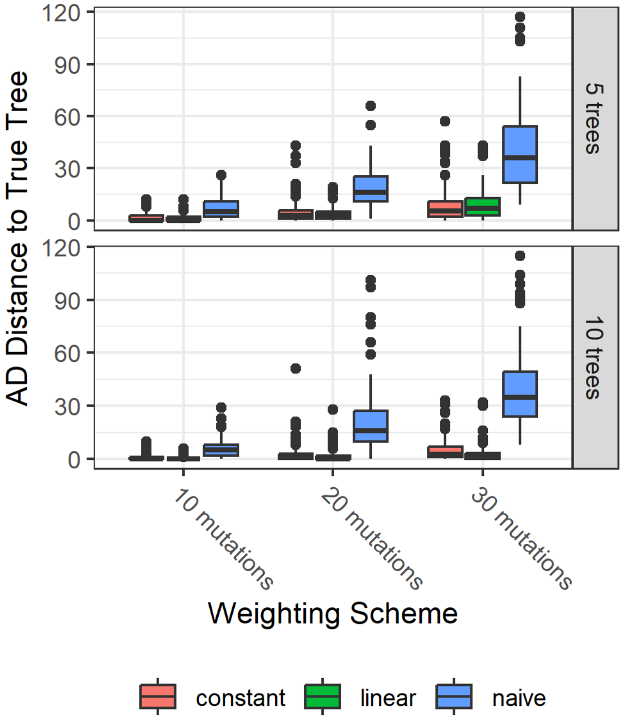

Fig. 2. For each trial, a different weighting scheme was used on the input trees for the TuELiPmodel, and the AD distance from the consensus tree to the true tree is calculated.

different mutation ordering, the tree returned from the center weights follows the same topology of the B-SCITE tree but with the mutations *TTN*, *HIPK4*, and *OAZ3* collapsed as well as mutations *CAMSAP1* and *SMOC1* in a single cluster.

We observe in Figure 11 (right) that the center section of the triangle plot is much more heterogeneous with different consensus trees output, compared to that of the ALL data we just showed. This may be due to the densely clustered format of the bulk sequencing tree, and also these three trees seem more different to each other than the trees of the patient with ALL.

This experiment raises attention to the problem of weights assignment to these inferred trees from sequencing data. A slight change in the weight allocation can change the output, especially when input trees are dissimilar to each other.

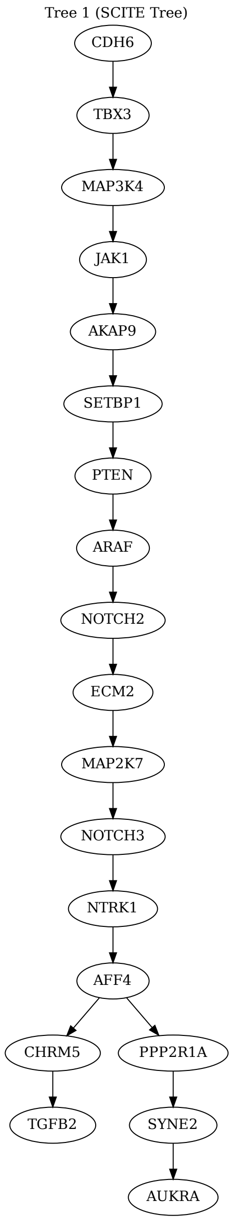

Fig. 3. Tree 1, equivalent to the tree inferred by SCITE (Jahn *et al.*, 2016), is inferred when $w(SCITE) > 0.5$.

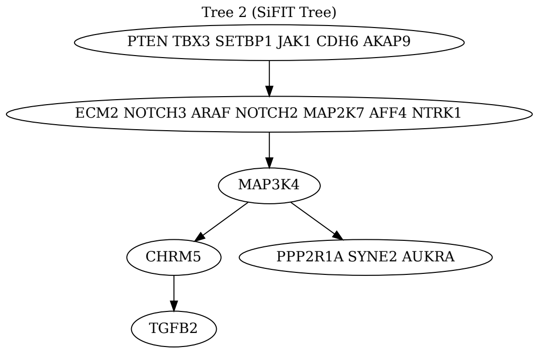

Fig. 4. Tree 2, equivalent to the tree inferred by SiFIT (Zafar *et al.*, 2017), is inferred when $w(SiFIT) > 0.5$.

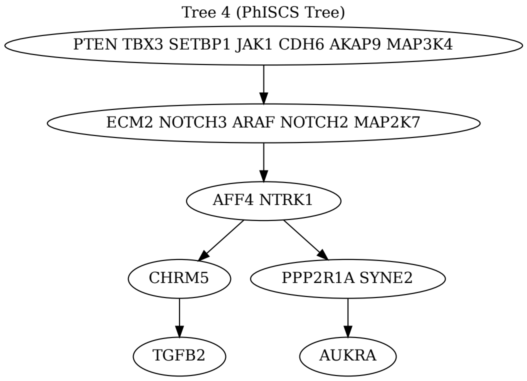

Fig. 6. Tree 4, equivalent to the tree inferred by PhISCS (Malikic *et al.*, 2019b), is inferred when the trees are equally weighted at $w(PhISCS) = \frac{1}{3}$, $w(SiFIT) = \frac{1}{3}$, and $w(SCITE) = \frac{1}{3}$.

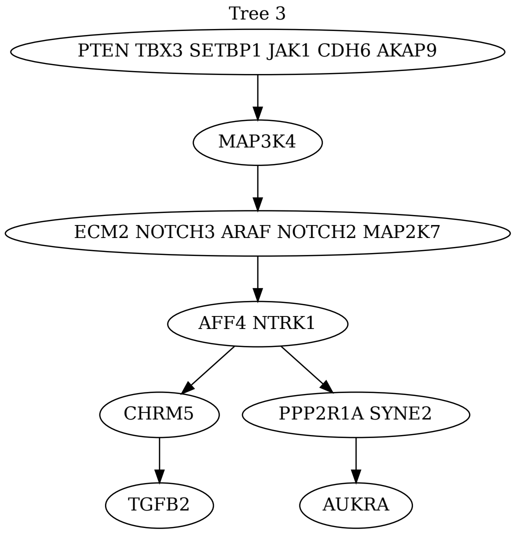

Fig. 5. Tree 3, is inferred when $w(PhISCS) = 0.2$, $w(SiFIT) = 0.4$, and $w(SCITE) = 0.4$.

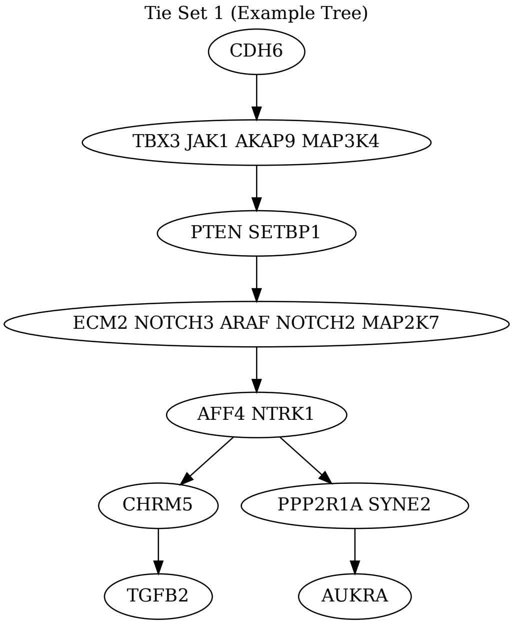

Fig. 7. This is an example of one of many trees from Tie Set 1 which are inferred when $w(SCITE) = 0.5$.

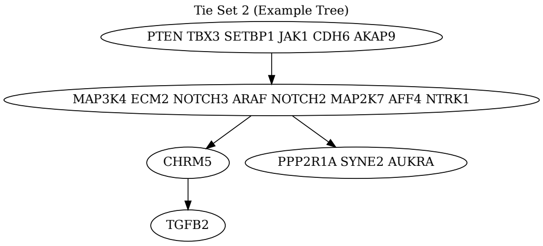

Fig. 8. This is an example of 1 of 16 trees from Tie Set 2 which are inferred when $w(SiFIT) = 0.5$ and $0 < w(PhISCS) < 0.5$.

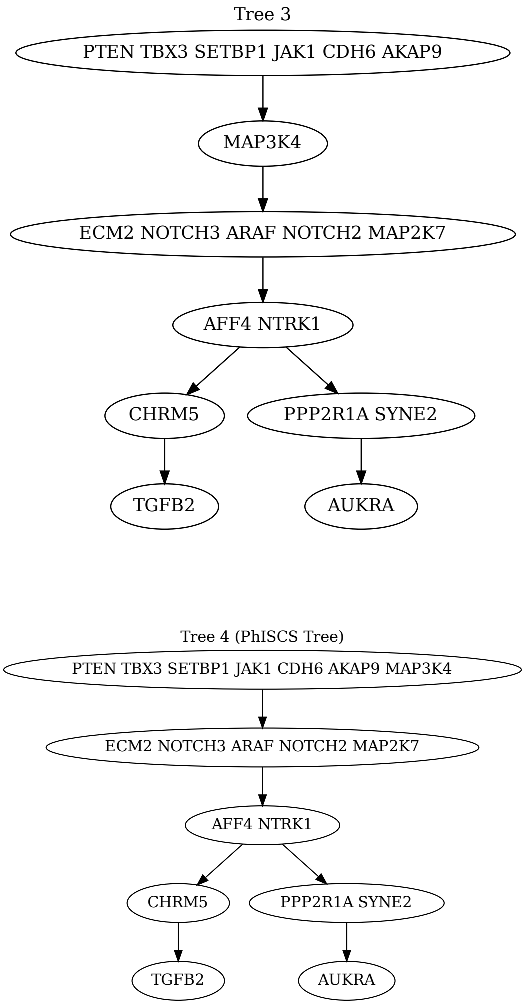

Fig. 9. Tree 3 and tree 4 are the two trees in Tie Set 3 which are inferred when $w(PhISCS) = 0.3$, $w(SiFIT) = 0.4$, and $w(SCITE) = 0.3$.

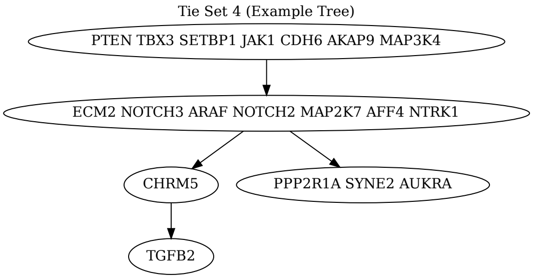

Fig. 10. This is an example of 1 of 20 trees from Tie Set 4 which are inferred when $w(PhISCS) = 0.5$, $w(SiFIT) = 0.5$, and $w(SCITE) = 0$.

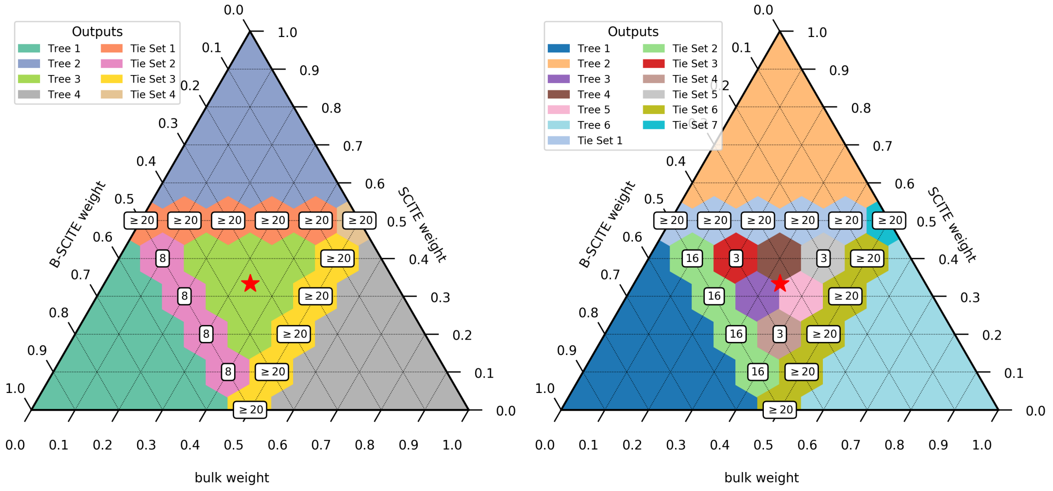

Fig. 11. The weights of each input tree from two patients were varied, while still adding up to 1, and used as input to TuELiP. The number of ties are present when there is more than one optimal tree for the given weight. The red star denotes the center of the triangle in which all three trees were given a weight of $\frac{1}{3}$. (Left) three input trees inferred by Malikic *et al.* (2019a) based on data from a study on a patient with ALL performed by Gawad *et al.* (2014) were used as inputs. (Right) three input trees inferred by Malikic *et al.* (2019a) based on data from a study on a patient with TNBC performed by Wang *et al.* (2014) were used as inputs.

References

Aguse, N. *et al.* (2019). Summarizing the solution space in tumor phylogeny inference by multiple consensus trees. *Bioinformatics*, **35**(14), i408–i416.

DiNardo, Z. *et al.* (2019). Distance measures for tumor evolutionary trees. *Bioinformatics*, **36**(7), 2090–2097.

Fu, X. and Schwartz, R. (2021). Contreedp: A consensus method of tumor trees based on maximum directed partition support problem. In *2021 IEEE International Conference on Bioinformatics and Biomedicine (BIBM)*, pages 125–130. IEEE.

Gawad, C. *et al.* (2014). Dissecting the clonal origins of childhood acute lymphoblastic leukemia by single-cell genomics. *Proceedings of the National Academy of Sciences*, **111**(50), 17947–17952.

Govek, K. *et al.* (2020). Graphyc: Using consensus to infer tumor evolution. *IEEE/ACM Transactions on Computational Biology and Bioinformatics*.

Jahn, K. *et al.* (2016). Tree inference for single-cell data. *Genome biology*, **17**(1), 1–17.

Jiao, W. *et al.* (2014). Inferring clonal evolution of tumors from single nucleotide somatic mutations. *BMC bioinformatics*, **15**(1), 1–16.

Malikic, S. *et al.* (2019a). Integrative inference of subclonal tumour evolution from single-cell and bulk sequencing data. *Nature communications*, **10**(1), 1–12.

Malikic, S. *et al.* (2019b). Phiscs: a combinatorial approach for subperfect tumor phylogeny reconstruction via integrative use of single-cell and bulk sequencing data. *Genome research*, **29**(11), 1860–1877.

Wang, Y. *et al.* (2014). Clonal evolution in breast cancer revealed by single nucleus genome sequencing. *Nature*, **512**(7513), 155–160.

Zafar, H. *et al.* (2017). Sifit: inferring tumor trees from single-cell sequencing data under finite-sites models. *Genome biology*, **18**(1), 1–20.
